# Role of Serum Uric Acid and Ferritin in the Development and Progression of NAFLD

**DOI:** 10.3390/ijms17040548

**Published:** 2016-04-12

**Authors:** Rosa Lombardi, Giuseppina Pisano, Silvia Fargion

**Affiliations:** Department of Pathophysiology and Transplantation, IRCCS “Ca’ Granda” IRCCS Foundation, Poiliclinico Hospital, University of Milan, Centro delle Malattie Metaboliche del Fegato, Milan 20122, Italy; rosalombardi@hotmail.it (R.L.); pinaz81@hotmail.com (G.P.)

**Keywords:** SUA, liver damage, fibrosis, NASH, serum markers, oxidative stress, insulin resistance, metabolic syndrome

## Abstract

Nonalcoholic fatty liver disease (NAFLD), tightly linked to the metabolic syndrome (MS), has emerged as a leading cause of chronic liver disease worldwide. Since it is potentially progressive towards non-alcoholic steatohepatitis (NASH) and hepatic fibrosis, up to cirrhosis and its associated complications, the need for predictive factors of NAFLD and of its advanced forms is mandatory. Despite the current “gold standard” for the assessment of liver damage in NAFLD being liver biopsy, in recent years, several non-invasive tools have been designed as alternatives to histology, of which fibroscan seems the most promising. Among the different serum markers considered, serum uric acid (SUA) and ferritin have emerged as possible predictors of severity of liver damage in NAFLD. In fact, as widely described in this review, they share common pathogenetic pathways and are both associated with hepatic steatosis and MS, thus suggesting a likely synergistic action. Nevertheless, the power of these serum markers seems to be too low if considered alone, suggesting that they should be included in a wider perspective together with other metabolic and biochemical parameters in order to predict liver damage.

## 1. Introduction

Nonalcoholic fatty liver disease (NAFLD), tightly linked to metabolic syndrome (MS), has emerged as a leading cause of chronic liver disease worldwide with a rapidly growing prevalence in the general population, ranging between 20% and 30%, and paralleling the epidemics of obesity and type 2 diabetes mellitus (T2DM) all over the world [1,2]. NAFLD encompasses a clinical-pathologic spectrum of liver diseases ranging from simple steatosis to nonalcoholic steatohepatitis (NASH), the more aggressive form of NAFLD, which can progress to cirrhosis and its associated complications [3,4].

Unfortunately, the only validated method to diagnose NASH, the potentially evolving form of NAFLD, is liver biopsy. Nonetheless, this procedure is limited by intra and inter-observer variability, sampling errors and invasiveness, thus letting impossible its feasibility in such a large number of patients with NAFLD. Several scores have been designed in the attempt to diagnose NASH and fibrosis stage without histological data, but the debate on their real utility is still ongoing [5]. Fibroscan is emerging as a reliable tool to identify fibrosis in a non-invasive way, but still the large “grey area” of its results does not allow one to discriminate the entity of fibrosis in a large portion of patients with NAFLD [6].

During the last few years, among the several parameters evaluated as possible predictors of NAFLD, serum uric acid (SUA) and ferritin have emerged. In fact, increasing evidence has shown that SUA levels as well as high ferritin are associated with the metabolic insulin resistance syndrome, higher body fat content and more severe liver damage.

## 2. Uric Acid

Serum uric acid (SUA) is a product of purine metabolism in humans and originates from hypoxanthine after a double enzyme catalysis by xanthine oxidase (XO) in the liver. Its production is regulated by the endogenous (nucleoproteins originating from cellular metabolism) and exogenous (dietary) precursor proteins delivered to the liver, whereas its excretion is controlled by the kidneys through renal plasma flow, glomerular filtration and proximal tubular exchange. Therefore, an impairment in this balance, caused by either an over generation of uric acid, like in MS and diets rich in fructose and purines or by a reduction in its excretion, as in acute renal failure or consequent to some drugs (ciclosporin, ethambutol, pyrazinamide, and cytotoxic chemotherapy), can lead to high SUA levels [7,8].

## 3. Serum Uric Acid and Metabolic Syndrome Clinical Manifestations

SUA is the most common and well-studied risk factor for developing gout. In addition, beyond contributing to the pathogenesis of gout, arthritis, and chronic nephropathy, hyperuricemia is associated with the so-called “cardio-metabolic diseases” including cardiovascular disease and all the metabolic diseases associated with MS [9]. Several studies reported a significantly higher prevalence of MS (up to 60%) and its components such as hypertension, hyperinsulinemia, hypertriglyceridemia and diabetes in the hyperuricemic population, suggesting that hyperuricemia might be an indicator for early diagnosis of MS and of its different clinical manifestations [10,11,12]. Moreover, a meta-analysis of prospective cohort studies provided strong evidence that a high level of SUA is a risk factor for developing T2DM in middle-aged and older people, independently of other established metabolic risk features [13].

## 4. Serum Uric Acid and Nonalcoholic Fatty Liver Disease (NAFLD)

Lonardo *et al.* [14] firstly described an association between NAFLD and serum uric acid levels in a small case-control study of Italian patients with ultrasound-diagnosed NAFLD.

The relationship between SUA and NAFLD was then confirmed in cross-sectional and prospective studies in which SUA resulted to be an independent risk factor for NAFLD [15,16]. More recently in two different meta-analyses of prospective studies including very large numbers of participants, it was shown a significant higher risk of NAFLD in subjects with higher SUA compared to those with lower levels. A linear dose-response effect between SUA and NAFLD was reported with each 1 mg increase of SUA leading to 21% rise in NAFLD risk [17,18]. Moreover, in patients with established coronary artery disease, hyperuricemia was reported to be a potent predictor of mortality in overweight or obese patients in whom liver steatosis was highly prevalent [19].

The mutual relationship between NAFLD and SUA was shown in another study aimed at exploring the causal relationship and underlying mechanisms linking NAFLD and hyperuricemia. By analyzing prospectively a cohort of 5541 patients, NAFLD resulted strongly associated with the risk of developing hyperuricemia over a period of seven years. In a second part of the same study, xantine oxidase was demonstrated to be the mediator of this relationship through the activation of the ucleotide-binding oligomerization domain-like (NOD-like) receptor family pyrin domain containing 3 (NLRP3) inflammasome [20] in both in HepG2 cells and mice, as explained in the next pharagraph. However, a major limitation of these study designs is their inability to show the biological mechanisms underpinning the association between SUA and NAFLD. Furthermore, experimental animal models supporting this association do not always mirror human biology.

Interestingly, Sirota *et al.* [21] examined the association between SUA levels and NAFLD in a large population-based study from the United States including 10,732 non-diabetic adults who participated in the National Health and Nutrition Examination Survey 1988–1994. The Authors found that the odds ratio for NAFLD was significantly higher in patients with the highest SUA values (3rd and 4th quartiles) compared to subjects in the lowest quartiles. In addition, after adjusting for the known risk factors, uric acid (4th quartile) remained significantly associated with NAFLD. Thus, they concluded that elevated SUA level is independently associated with ultrasound-diagnosed NAFLD and with increasing severity of NAFLD as evaluated by ultrasonography. These data were in line with previous results by Petta *et al.* [22] obtained in a group of patients with histologically proven NAFLD. They had demonstrated that hyperuricemia was associated with histological features of liver disease, representing an independent risk factor for higher grade of steatosis, lobular inflammation and higher NAFLD Activity Score (NAS), the histological score routinely used for the diagnosis and grading of NASH. Thus, these data confirm and extend the results obtained in Asiatic subjects also to Caucasian patients, consolidating the relationship between NAFLD and SUA.

Finally, Afzali *et al.* [23], on the basis of the observation that elevated SUA levels strongly reflect and may even cause oxidative stress, insulin resistance (IR), and MS, and that experimental, and in *in vitro* models indicate that uric acid is able to induce inflammatory responses, all known risk factors involved in the pathogenesis and in the progression of liver disease of different etiology, addressed the question whether the baseline SUA level was associated with the incidence of hospitalization or death due to cirrhosis. These authors analyzed 5518 participants from the first National Health and Nutrition Examination Survey during a mean follow-up of 12.9 years (range 5.4–21 years) and demonstrated that subjects with the higher uric acid values had a higher risk of cirrhosis related hospitalization or death even after adjustment for important causes and risk factors of chronic liver disease. In addition, patients with higher SUA levels had a greater probability of elevated serum ALT and GGT. They suggested that the negative effect of SUA was mediated by the induction by uric acid of endothelial dysfunction, IR, oxidative stress and systemic inflammation, which are known risk factors for the development and progression of liver disease of different etiology. However, despite this fascinating hypothesis, a major limitation of these results obtained in clinical studies is that the direct demonstration in patients with NAFLD of the mechanisms underpinning the negative effect of SUA is still missing.

## 5. Relationship between Uric Acid and NAFLD/Metabolic Syndrome: Possible Mechanisms

**Figure 1:**
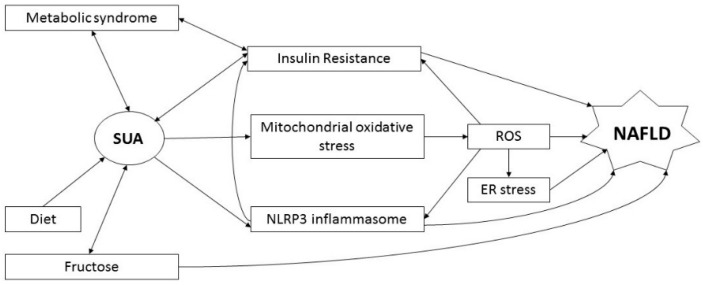
Pathogenetic pathways of the association between serum uric acid and NAFLD. Abbreviations: SUA, serum uric acid; ROS, reactive oxygen species; ER, endoplasmic reticulum; NAFLD, non-alcoholic fatty liver disease.

Accumulating clinical evidence suggests that hyperuricemia is strongly associated with MS/NAFLD, and abnormal glucose metabolism and IR, as well as oxidative stress and NLRP3 inflammasome involvement, have been pointed out as possible linking conditions [11,24]. The possible interactions of the different mechanisms involved are schematically depicted in Figure 1.

Furthermore, a very recent study by a Chinese group, has focused on the progression of NAFLD in hyperuricemic subjects, showing a key role of perturbations of phospholipases, purine nucleotide degradation and Liver X receptor/retinoid X receptor. In particular, they demonstrated an increase in oxidative stress and IR driven by an upregulation of phospatidic acid and cholesterol ester metabolism and a downregulation of the acid uric precursor, namely inosin [25].

### 5.1. Interaction between Uric Acid and Insulin

Insulin acts on the proximal renal tubule fostering acid uric reabsorption and increasing renal cellular metabolism, thus leading to hyperuricemia. Indeed, elevated SUA levels may prompt the development of IR by reducing endothelial nitric oxide (NO) bioavailability and supply to cells [26].

In addition, in an experimental model, mice fed with hyperuricemia-inducing diet (HUA) presented significantly lower insulin sensitivity and impaired glucose metabolism compared to those with a standard diet, as well as higher levels of both serum and intrahepatic triglycerides. In particular, hyperuricemia inhibited a protein kinase B (AKT) response to insulin by decreasing its phosphorylation and conversely increasing the phosphorylation of the insulin receptor substrate-1 (IRS1) in liver, muscle and fat tissue, thus fostering the onset of IR. This effect seems to be secondary to uric acid induced radical oxygen species (ROS) and activation of the NLRP3 inflammasome [27,28]. These data were confirmed also in HepG2 cell cultures exposed to different concentrations of uric acid. Not surprisingly, administration of probenecid, a uric acid transport inhibitor into cells, or the antioxidant *N*-acetylcisteine, diminished intracellular triglycerides accumulation and improved insulin-signaling.

### 5.2. Uric Acid and Lipid Metabolism

Beyond hyperinsulinemia, uric acid is responsible of mitochondrial oxidative stress [29], sterol regulatory element-binding protein 1 (SREBP-1) activation induced by endoplasmic reticulum (ER) stress [30] and NLRP3 inflammasome involvement [31], all causative factors of lipid metabolism impairment.

Moreover, evidence suggests that uric acid could originate from fructose metabolism, which is well known for inducing hepatic steatosis being directly metabolized to triglycerides in the liver [32], and be responsible for mitochondrial oxidative stress. In turn, SUA amplifies the lipogenic effect of fructose by upregulating its metabolic enzymatic reactions [33]. In cultured HepG2 cellular lines exposed to fructose, increased intracellular levels of uric acid and triglycerides were registered. Interestingly, allopurinol effectively prevented the formation of uric acid after exposure to fructose [29].

### 5.3. Mitochondrial and Endoplasmic Reticulum (ER) Oxidative Stress

Oxidative stress plays a key-role in steatosis induced by uric acid. In the study by Lanaspa *et al.* [29], cellular exposure to high SUA levels determined mitochondrial oxidative stress with generation of ROS by nicotinamide adenine dinucleotide phosphate (NADPH) oxidase. As a result, the activity of aconitase, an enzyme involved in the acid citric circle, was markedly reduced leading to accumulation of citrate, a substrate for hepatic *de novo* lipogenesis and subsequent intracellular fat generation.

Furthermore, ROS production promotes ER stress, which is determinant of fat accumulation in steatosis. In fact, alterations in its homeostasis have been demonstrated in human HepG2 cells and mice models of fatty liver [34,35]. ER is a site of protein folding and production of lipids and sterols. If a perturbation in this compartment occurs, misfolded and unfolded proteins accumulate and activate the unfolded protein response (UPR) signaling pathways, which regulate hepatic lipid metabolism and promote fat accumulation in the liver because of the expression of genes encoding for lipogenic enzymes driven by the transcriptional factor SREBP-1c. Uric acid has been shown to induce the expression of unfolded response protein (URP)-inducible and to increase the cleavage of SREBP-1c into the mature form and its nuclear translocation, thus enhancing the *de novo* lipogenesis. This data has been shown in both HepG2 cells and primary mice hepatocytes [30].

Despite these data, acute elevations seem to provide antioxidant protection, and uric acid contributes >50% of the antioxidant capacity of our organism. In fact, it has a direct effect on the inhibition of free radicals, protecting the cell membrane and DNA. The antioxidant activity of SUA also occurs in the brain, being a protector for several disease such as multiple sclerosis and neurodegenerative disease, as well as cardiac and renal toxicity [36]. Thus, an eventually beneficial action could be speculated also on the liver.

In addition, there is still no consensus if uric acid is a protective or a risk factor; however, it seems that the quantity and the duration of the concentration of the uric acid in the blood is essential for this answer, possibly being the acute increase in its protective levels whereas chronic elevated levels are dangerous.

### 5.4. The Ucleotide-Binding Oligomerization Domain-Like (NOD-Like) Receptor Family Pyrin Domain Containing 3 (NLRP3) Inflammasome

Another factor which has been reported to be strongly involved in the pathogenesis of uric acid toxicity is NLRP3 inflammasome, an intracellular multiprotein complex that is assembled and activated by pathogen-associated and damage-associated molecular patterns with subsequent production of pro-inflammatory and pro-fibrotic cytokines (IL-1β and IL-18). It plays a central role in obesity and IR and has been involved in dyslipidemia and lipid accumulation in hepatocytes [28,31]. The NLRP3 inflammasome is activated by uric acid, both directly and indirectly through ROS production [37] and recent evidence has demonstrated that it contributes to hepatic steatosis and insulin resistance in a murine model [28]. This suggestion was confirmed in cultured HepG2 and L02 cellular lines, where the NLRP3 inflammasome knock-down cells decreased the uric acid-induced hepatic free fatty acids (FFAs) accumulation [31].

In conclusion, SUA is able to regulate lipid production and to foster the onset of metabolic disorders and NAFLD through multifaceted pathways. Thereby, evidence is accumulating on the benefit of lowering SUA levels in NAFLD by using drugs commonly employed in the treatment of hyperuricemia, like allopurinol or probenecid.

## 6. Ferritin

Hyperferritinemia is a frequent finding in the general population, is detected in 30%–40% of the patients with MS/NAFLD, and has been suggested as a marker of severity of the disease.

The difficulty in the interpretation of increased ferritin is related to the multiple causes that can lead to its increase, initially identified as marker of iron overload, following the increase of transferrin saturation, and also in the presence of severe hepatic necrosis. Furthermore, other more frequent causes need to be considered, namely the presence of inflammation, since ferritin behaves as a protein of acute phase and it can also be induced in the setting of systemic inflammation, like in rheumatologi, infectious or neoplastic diseases, and alcohol abuse, where ferritin levels rapidly decrease with alcohol abstinence. However, enlarging the most common cause of hyperferritinemia identified in the last years is the presence of the MS, to which NAFLD is frequently associated.

## 7. Ferritin and Metabolic Syndrome Clinical Manifestations

Hyperferritinemia is detected in about one-third of patients with NAFLD and the MS and its levels seem to be directly correlated with the severity of IR [38,39].

The first reports on the relationship between ferritin, IR/T2DM and the MS study in Europe were published in the 1990s. In 1998, Ford *et al.* [40] reported the results of a case-control study in Europe, demonstrating that subjects with hyperferritinemia had a 2.4-fold higher risk of developing T2DM. In addition, Salonen [41] showed in a prospective study that increased ferritin levels precede the development of diabetes and Kim obtained the same results in a very large cohort of Korean subjects [42]. In addition, cross-sectional studies found that elevated ferritin levels were associated with central obesity [43], hypertension [44], and dyslipidemia [45], all manifestations of the MS. Moreover, Iwasaki highlighted an association between serum ferritin, visceral fat and subcutaneous adiposity and suggested that serum ferritin concentration may be a useful indicator of systemic fat content and degree of IR [46]. In addition, Alam *et al.* [47] demonstrated that obesity led to hyperferritinemia irrespective of actual body iron story, advocating a state of subclinical inflammation responsible for high levels of ferritin.

Others demonstrated in population-based studies that moderate to markedly increased ferritin concentrations represent a biological biomarker predictive of early death in a dose-dependent manner [48]. Thus, even if in this study, information on the presence of liver steatosis was lacking, it is very likely that ferritin may be a predictor of early death also in the setting of NAFLD.

## 8. Ferritin and NAFLD

The tight link between ferritin and insulin dysregulation was shown by Fernandez-Real [49], who proposed ferritin as a marker of IR. Zelber-Sagi *et al.* [50]demonstrated that among different metabolic features, insulin was the strongest predictor of increased serum ferritin levels and that the association between serum ferritin and MS was mediated by NAFLD.

A French group coined the term of “*dysmetabolic iron overload syndrome*” (DIOS), to indicate subjects with increased ferritin levels, with normal or only mildly increased transferrin saturation, in the presence of liver steatosis, IR and two or more components of the MS, along with moderate hepatic iron accumulation with the typical pattern of mixed parenchymal and mesenchymal iron deposition [51]. However, it was also observed that several patients with NAFLD, IR and manifestations of MS may have increased ferritin even in the absence of increased iron stores.

In addition, Kim *et al.* [42] reported that serum ferritin levels predict incident non-alcoholic fatty liver disease in healthy Korean men.

## 9. Relationship between Ferritin and NAFLD/Metabolic Syndrome: Possible Mechanisms

Numerous data demonstrate that hepatic iron accumulation could elicit the onset of metabolic imbalance and liver damage and figure out the DIOS or more recently called “insulin-resistance associated with iron overload syndrome”.

The liver has a central role in iron metabolism as it is the principle source of hepcidin, the regulatory peptide hormone of iron homeostasis. In fact, in response to several stimuli, like excessive iron deposits, inflammatory signals (IL-6) or ER-stress, hepcidin is overexpressed and determines a reduction in iron intestinal absorption and an increase in iron retention from macrophage and hepatocytes [52,53]. In addition, hepatocyte necrosis, with subsequent erythrophagocytosis by macrophages, and the systemic inflammatory state induced by obesity and NAFLD itself, may predispose individuals to increased hepcidin levels.

Many mechanisms linking iron and liver damage have been described. Firstly, iron, once accumulated in the liver, causes oxidative stress through the Fenton and Haber–Weiss chemistry with production of ROS and damage to membranes, proteins and DNA. Secondly, ferritin itself, which is the expression of iron storage in the liver, behaves as a real pro-inflammatory cytokine directly activating the hepatic stellate cells via Nuclear Factor κB (NFκB) cascade and inducing fibrogenesis [54]. Nevertheless, the role of hepatic iron and progression of liver disease is still to be fully elucidated.

In addition, very recent data suggest a possible role of splenic iron accumulation in promoting liver damage. However, these results need further confirmations [55].

### 9.1. Pathogenesis of DIOS (Dysmetabolic Iron Overload Syndrome)

**Figure 2:**
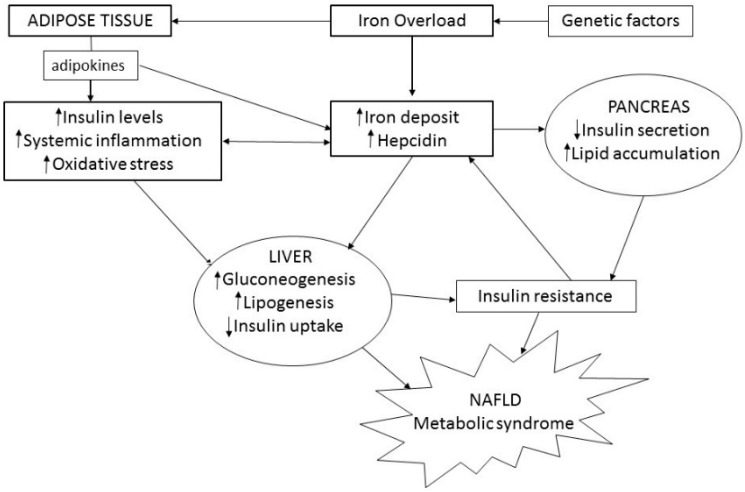
Pathogenetic pathways of the association between ferritin and NAFLD. Up arrow in the boxes: increase; down arrow in the boxes: decrease.

Several explanations for the correlation between high ferritin levels and NAFLD have been proposed, namely IR, erythrophagocytosis by hepatic macrophages and dysregulation of proteins and pathways involved in iron homeostasis. Among the latter, hepcidin seems to have a key role in iron accumulation in NAFLD [56,57], as increased levels of this peptide have been detected in these patients [58,59], as well in the paediatric NAFLD population [60]. Furthermore, an influence of genetic factors has been considered, in particular the heterozygosis state of β-thalassemia and mutations in the HFE gene responsible for hereditary hemochromatosis (HH) [38,61,62] (Figure 2).

### 9.2. Hyperferritinemia and Insulin-Resistance

The relationship between hyperferritinemia and IR seems to be mutual. In fact, early *in vitro* studies have suggested that insulin might determine a rapid and marked stimulation of iron uptake by fat cells, by a redistribution of transferrin receptors from an intracellular membrane compartment to the cell surface [49]. On the other hand, systemic iron overload may prompt the onset of diabetes mellitus (DM) consequent to an impairment in pancreatic β-cells function due to intra-parenchymal iron deposition. In fact, because of oxidative stress, β-cells are less sensitive to glucose stimulation and die by to apoptosis with consequent reduction in insulin production [63].

The effect of iron overload on glucose metabolism has been investigated in animal models. In a study by Choi *et al.* [64], mice fed with a standard diet enriched in iron, presented higher levels of ferritin, hepcidin and inflammatory cytokines, as well as a higher degree of IR and metabolic dysregulations, mainly driven by an overexpression of genes involved in either gluconeogenesis or lipogenesis. These features were exasperated in mice fed with high fat diet (HFD) enriched with iron, suggesting a synergistic effect of fat and iron. The authors have speculated that insulin stimulates ferritin synthesis via inflammatory pathways and enhances hepcidin expression. On the other hand, iron interferes with insulin inhibition of glucose production by the liver and decreases the hepatic extraction and metabolism of insulin, leading to peripheral hyperinsulinemia. These data were confirmed by Dongiovanni *et al.* [65], who showed that an iron-enriched diet in mice led to the development of IR, probably due to the secretion of adipokines by the visceral adipose tissue consequent to iron accumulation.

Recent data by Vecchi *et al.* [66] further explored the relationship between glucose and iron metabolism and showed a new regulatory pathway in iron homeostasis driven by gluconeogenic stimuli and with the major actors being hepcdin and PPARGC1A, a transcriptional coactivator of genes involved in gluconeogenesis. Therefore, in conditions like NAFLD, obesity and T2DM, persistently activated gluceoneogenesis may result in overstimulation of hepcidin and iron accumulation.

The interplay between iron and insulin has been also confirmed by experimental data that showed how iron depletion could elicit an over expression and higher affinity of insulin receptors, as well as an increase in the expression of molecules involved in the intracellular signal cascade activated by insulin receptors and of genes involved in glucose uptake [57].

### 9.3. Hyperferritinemia and Adipose Tissue

The adipose tissue behaves as an endocrine organ, which under a condition of chronic inflammation, as in NAFLD, releases adipokines in the bloodstream, thus altering glucose and iron homeostasis and may determine a condition of subclinical inflammation itself [47,67]. Many adipokines play a central role in this scenario, namely adiponectin, leptin and resistin [57]. Adiponectin, which is an anti-steatotic and anti-inflammatory adipokine, is reduced in dysmetabolic conditions like NAFLD, IR and T2DM, and seems to predict the severity of liver inflammation and fibrosis. In fact, it has the capability of inducing the transcription of key genes in iron metabolism, like the hemeoxygenase-1 (HO-1), determining lower iron levels in hepatocytes, thus preventing apoptosis. Conversely, leptin, an adipokine involved in the control of food intake and energy consumption, seems to upregulate hepcidin synthesis, thus contributing to DIOS pathogenesis.

Finally, resistin is able to either impair glucose tolerance or reduce glucose uptake from muscular tissue or induce an inhibitor of insulin signaling namely SOCS3 (Suppressor of cytokine signaling 3), thus eliciting a condition of IR.

In line with this are the results by Beckry *et al.* [68] who have shown the ectopic expression of hepcidin in white adipose tissue of obese individuals and that of leptin, usually increased in obese subjects, was able to enhance hepcidin mRNA *in vitro*. In addition, Green *et al.* [69] have demonstrated how isolated primary rat adipocytes exposed to iron become insulin-resistant decreasing insulin mediated glucose transport and fostering lipolisis. On the other hand, a “portal vein theory” has been proposed and comprises the concept that visceral adipose tissue and/or the gut release into the portal vein increasing amounts of FFAs and pro-inflammatory factors, which, in turn, reach the liver and contribute to the onset of hepatic IR and steatosis. However, further studies are needed for a better comprehension of this casual link [70].

## 10. Hyperferritinemia and Severity of Liver Damage in NAFLD

Iron and ferritin have been hypothesized to foster the progression of organ damage, including hepatic and cardiovascular diseases.

In 2001, our group showed that hyperferritinemia with normal transferrin saturation was a hallmark of a glucose/lipid metabolism disorder and, when associated with multiple metabolic abnormalities and iron overload, identified patients at risk for NASH. Interestingly, we observed that patients in whom ferritin remained elevated despite lifestyle modifications (diet, weight loss, physical activity) differed from those whose ferritin normalized, presenting the former a more severe liver disease. We hypothesized that the increase of ferritin possibly reflected a synergistic induction of its synthesis because of increased iron stores, hepatic steatosis and subclinical inflammation. In contrast, when the increase in serum ferritin was a consequence only of altered lipid metabolism, it was reversible with diet and unrelated to iron stores [71].

Since then, several other studies analyzed the relationship between ferritin, iron overload and severity of liver damage in patients with NAFLD. Bugianesi *et al.* [38] demonstrated that increased ferritin levels are markers of severe histologic damage, but not of iron overload and that iron burden and HFE mutations do not contribute significantly to hepatic fibrosis in the majority of patients with NAFLD. Manousou *et al.* [72] evaluated in 111 NAFLD patients the relationship between serum ferritin and features of MS with respect to histological inflammation and/or fibrosis. Interestingly, ferritin resulted a good predictor of advanced liver disease, with respect to both NASH and fibrosis. In addition, Kowdley *et al.* [73] demonstrated that elevated serum ferritin is an independent predictor of histologic severity and advanced fibrosis among patients with NAFLD. He found in a cohort of 628 biopsy-proven NAFLD with hyperferritinemia that ferritin, besides being significantly associated with markers of liver damage (elevated serum ALT, AST and decreased platelets) and of iron overload (iron, transferrin-iron saturation and iron stain grade), was associated with more severe histologic features of NAFLD, including steatosis, hepatocellular ballooning, increased NAFLD Activity Score (NAS) and diagnosis of NASH. In addition, ferritin was also independently associated with advanced hepatic fibrosis and with higher NAS, the latter even among patients without hepatic iron deposition. The authors concluded that serum ferritin was useful to identify NAFLD patients at risk for NASH and advanced fibrosis.

These data were confirmed in a cohort of 108 Korean biopsy proven NAFLD patients in whom a positive correlation between ferritin level, metabolic alterations, liver fibrosis and NASH was found. Nevertheless, the association between ferritin and histology resulted weaker compared to another serum marker resulting from hepatocytes apoptosis, namely fragmented cytokeratin-18 (CK-18) [74].

Conversely, Angulo *et al.* [75] retrospectively analyzed in 1404 NAFLD patients the accuracy of serum ferritin in determining the presence and severity of liver fibrosis, and whether combining non-invasive fibrosis scoring systems with serum ferritin analysis could increase the accuracy of those tests. Although serum levels of ferritin correlated with more-severe liver fibrosis; however, either the performance of ferritin resulted unsatisfactorily for any grade of fibrosis or the accuracy of the non-invasive scores did not change with inclusion of serum ferritin. On the basis of adjusted multiple logistic regression analysis, they concluded that serum ferritin levels alone had a low level of diagnostic accuracy for the presence or severity of liver fibrosis in patients with NAFLD.

Similar results were reported by Yoneda *et al.* [76], who analyzed 1201 biopsy-proven NAFLD patients previously enrolled into the Japan Study Group of NAFLD and belonging to a large Japanese cohort database of NAFLD patients. By comparing serum ferritin levels and hepatic histology, the authors showed that ferritin increased with increasing histological grade of steatosis, lobular inflammation and ballooning and that at multivariate analyses it was independently associated with steatosis grade and fibrotic stage. However, ferritin showed a suboptimal performance as predictive test of any degree of liver fibrosis, possibly because several other factors including sex and metabolic features could have interfered. The conclusion of the study was that serum ferritin had a low diagnostic accuracy for detecting fibrosis in NAFLD patients when considered alone.

Nevertheless, ferritin has also been included in serum panels in order to detect liver damage non-invasively. One of these is the NAFIC score, which relies on ferritin, insulin and type IV collagen serum levels and which has been tested in a cohort of 147 biopsy-proven NAFLD and validated in another cohort of 355 patients from nine hepatologic centers in Japan. A cut-off of two has been identified to diagnose the presence of NASH in NAFLD patients, with a sensitivity and a specificity of 63% and 83%, respectively. Later, a new modified NAFIC score was created including higher insulin values that presented a better diagnostic performance (sensitivity 74%, specificity 75% and Area under Receiving Operating Characteristic—AUROC 0.801) [77].

Another score which includes ferritin as a variable is the FibroMeter NAFLD score. It consists of a panel of serum markers and has been shown to have a high diagnostic accuracy for staging liver fibrosis. In particular, in a study of 235 NAFLD patients, it showed an AUROC of 0.94 for significant fibrosis (≥F2), 0.93 for severe fibrosis (F3), and 0.9 for cirrhosis [5,78].

In conclusion, increasing data aimed at pointing ferritin as possible predictive factor of liver damage are accumulating. Despite conflicting and still not conclusive results, it could be speculated that ferritin might be used as a surrogate marker, especially if combined with other metabolic and biochemical variables, to identify a more severe liver disease, even if with an intermediate sensitivity and specificity.

## 11. Does Hyperferritinemia Reflect Iron Overload?

In an attempt to clarify whether the increase in ferritin observed in patients with NAFLD reflects iron overload, studies were performed to define a possible association between ferritin and both liver siderosis and mutations in genes involved in iron metabolism. Interestingly, HFE mutations responsible for hereditary hemochromatosis resulted non significantly associated either to liver siderosis or hyperferritinemia and also liver damage did not result as being influenced by the presence of these mutations [62]. *Vice versa*, liver damage defined either by more severe fibrosis or presence of NASH, resulted as significantly associated with the presence of liver siderosis and β thalassemia traits [79]. However, the large majority of studies concluded that the increased ferritin values observed in patients with NAFLD reflect increased iron stores and acquired and genetic factors predisposing individuals to lipid and iron metabolism alterations in the presence of subclinical inflammation.

## 12. Iron Depletion in Patients with Hyperferritinemia, Metabolic Alterations and NAFLD

Iron is known for causing oxidative stress through the Fenton and Haber–Weiss chemistry with production of ROS and damage to membranes, proteins and DNA, thus being capable of inducing liver damage and fibrosis. Ferritin is the primary iron-storage protein and serum ferritin concentration has historically been used to predict severe fibrosis in chronic liver diseases.

Several studies showed that iron depletion therapy was followed by a reduction in plasma glucose and by an improvement of insulin sensitivity. Facchini *et al.* in 2002 [80] demonstrated in a small series of NAFLD patients, with and without increased ferritin levels, that iron removal in carbohydrate intolerant patients with clinical evidence of nonalcoholic fatty liver disease was able to improve insulin sensitivity in the short term (without changes in body weight). Fernandez-Real showed in a randomized trial that blood letting in high-ferritin T2DM improved insulin sensitivity and secretion [81]. In addition, Valenti *et al.* [82] demonstrated that iron depletion by venesection, in patients with moderate iron overload associated with NAFLD, determined a decrease of both IR and transaminases, as well as of ferritin levels.

Despite these encouraging data, confirmed also in following studies, the role of iron depletion in the improvement in liver histology and the natural history of liver disease is still under definition because of the lack of studies including a large number of patients.

## 13. Conclusions

NAFLD is recognized as the leading cause of chronic liver disease worldwide, and, in a percentage of cases, it is potentially progressive towards advanced fibrosis and severe complications. As a consequence, the need for predictive factors of NAFLD and especially of its progressive forms is mandatory. In recent years, SUA and ferritin have emerged as possible predictors of hepatic steatosis and liver damage. Interestingly, some studies have reported high SUA levels in patients with hyperferritinemia and *vice versa*, thus suggesting a mutual relationship and a synergistic action [83,84,85].

In fact, as extensively depicted in this review, SUA and ferritin share common pathogenic mechanisms, in particular oxidative stress and IR, and are associated with metabolic features, among the latter obesity and T2DM are the most important. Therefore, it could be speculated that both SUA and ferritin are the main actors in the multifaceted and complicated scenario of NAFLD and its dysmetabolic features.

However, given that the majority of studies are based on observational data, well-designed prospective studies including a large series of patients of different ethnicities are warranted before a definite role of SUA and ferritin in the pathogenesis of NAFLD can be established. In addition, it could be of interest to evaluate whether treating hyperuricemia and hyperferritinemia may lead to NAFLD improvement, and, in turn, whether regression of NAFLD is accompanied by a normalization of SUA and ferritin levels.
